# Genome Editing in Mouse Spermatogonial Stem/Progenitor Cells Using Engineered Nucleases

**DOI:** 10.1371/journal.pone.0112652

**Published:** 2014-11-19

**Authors:** Danielle A. Fanslow, Stacey E. Wirt, Jenny C. Barker, Jon P. Connelly, Matthew H. Porteus, Christina Tenenhaus Dann

**Affiliations:** 1 Department of Chemistry, Indiana University, Bloomington, Indiana, United States of America; 2 Department of Pediatrics, Stanford University, Stanford, California, United States of America; 3 Department of Biochemistry, UT Southwestern Medical Center, Dallas, Texas, United States of America; Nanjing Medical University, China

## Abstract

Editing the genome to create specific sequence modifications is a powerful way to study gene function and promises future applicability to gene therapy. Creation of precise modifications requires homologous recombination, a very rare event in most cell types that can be stimulated by introducing a double strand break near the target sequence. One method to create a double strand break in a particular sequence is with a custom designed nuclease. We used engineered nucleases to stimulate homologous recombination to correct a mutant gene in mouse “GS” (germline stem) cells, testicular derived cell cultures containing spermatogonial stem cells and progenitor cells. We demonstrated that gene-corrected cells maintained several properties of spermatogonial stem/progenitor cells including the ability to colonize following testicular transplantation. This proof of concept for genome editing in GS cells impacts both cell therapy and basic research given the potential for GS cells to be propagated *in vitro*, contribute to the germline *in vivo* following testicular transplantation or become reprogrammed to pluripotency *in vitro*.

## Introduction

Spermatogonial stem cells (SSCs) are at the foundation of spermatogenesis. Their maintenance is essential for the continuous production of spermatozoa throughout a male's reproductive lifetime. SSCs, like other adult stem cells, balance the process of self-renewal with the production of progenitor cells that will go on to differentiate. SSCs are also similar to many other stem cell types in that they are rare and difficult to identify definitively through expression of particular proteins [1], [2], [3]. Rather, rodent SSCs can be most strictly defined functionally based on their ability to home to a niche and colonize a recipient's testes following transplantation, and then undergo meiosis and differentiate into sperm [4], [5].

Following years of intensive effort by multiple laboratories, conditions were eventually discovered for enriching for SSCs and maintaining them essentially indefinitely *in vitro* [6], [7]. The cultured cells, termed “germline stem (GS) cells”, have properties of untransformed primary cells that can be propagated long-term because of the self-renewal of SSCs. Importantly, putative SSCs can also be identified and cultured *in vitro* from human testes, although the duration for which human SSCs can be kept *in vitro* remains controversial and conditions for long term culture need to be optimized [8], [9].

Given the robust nature of the rodent GS cell propagation system, we chose to model the process of *ex vivo* genome editing using mouse GS cells. We decided to test one of the more challenging genome editing approaches whereby homologous recombination is used to modify an existing mutation in the genome because this approach creates the precise modifications necessary for the most powerful research and therapy applications. For research purposes genome modification by homologous recombination (HR) greatly decreases the possibility of heterogeneous phenotypes from uncontrolled random integration; that is, with the latter, a transgene's expression may be variable or silenced depending on where it integrates. For therapeutic purposes HR is potentially safer because of the elimination of random insertions, which in certain settings have been shown to lead to cancer through the process of insertional oncogenesis [10]. While the frequency of HR with an exogenous DNA repair substrate in most cell types is too low to be therapeutically useful, the frequency can be increased by several orders of magnitude by introducing a double strand break (DSB) at the site in the chromosome to be modified [11], [12], [13], [14]. Creation of a DSB can be accomplished using custom designed nucleases, including zinc finger nucleases (ZFNs), TAL effector nucleases (TALENs) or RNA guided endonucleases [15], [16], [17], [18], [19], [20], [21], [22]. ZFNs and TALENs are chimeric proteins comprising a nuclease domain from the type II restriction enzyme Fok I and a DNA binding domain engineered to recognize a specific sequence.

ZFNs and TALENs have been demonstrated to stimulate homology directed repair of a DSB using an exogenous DNA repair substrate, or “gene targeting”, in a wide variety of contexts. For example, correction of a point mutation in interleukin 2 receptor, gamma (*IL2RG*) was accomplished in K562 cells, an immortalized myelogenous leukemia human cell line, as well as in primary human T-cells and human CD34+ cells [23], [24]. Also, ZFNs and TALENs have been shown to simulate gene targeting in mouse and human embryonic stem cells and induced pluripotent stem cells [25], [26], [27]. Still, genome engineering in the context of adult primary-like stem cells, which are likely to more closely resemble cells that will be used in therapy, is relatively unexplored. Moreover, the use of ZFN or TALEN stimulated HR to modify the genome in GS cells has not been described.

## Materials And Methods

### GS cell line derivation and culture

Cells used in gene correction experiments (designated MPG or F8R) were from mice that were homozygous for a previously described allele in which a mutated GFP reporter cassette was knocked in to the *ROSA26* locus [25]. F8R cells were derived from mice that were also homozygous for a mutation in the F8 gene (*F8^tm1Kaz^* obtained from The Jackson Laboratory, Bar Harbor, ME). GS cell lines were derived and maintained following published procedures with minor modifications as follows [6], [28], [29], [30]. Seminiferous tubules were mechanically separated, dissociated with dispase (BD Biosciences, San Jose, CA) and cultured overnight in Dulbecco's Modified Eagle's Medium/Nutrient Mixture F-12 Ham (Sigma, St. Louis, MO) with 10% FBS on a gelatin coated plate for removal of some somatic cells. Non-adhering cells were collected and cultured in F12GFB medium [28] on a feeder layer of mitotically inactivated DR4 MEFs. Use of F12GFB medium caused germ cells to grow as floating clusters while somatic cells adhered, allowing for enrichment of SSCs over the course of weeks through differential passaging by trituration [28]. Once SSCs were enriched the resulting cell lines (termed “GS cells”) were maintained on MEFs in Stem Pro based medium (“SPGF”, Stem Pro with GDNF and FGF2) containing 10 ng/mL GDNF and 10 ng/mL FGF2 and 19 other supplements as described [6], [30].

### Genome editing reagents

A ZFN expression plasmid (“M500”, ∼10 kB) was made by introducing a Ubiquitin C promoter and two previously characterized ZFNs, GFP-ZFN1 and GFP-ZFN2, separated by a T2A ribosomal skip sequence by standard molecular biology techniques into a lentiviral backbone generously provided by Dr. Eric Brown (Univ. of Pennsylvania) [25], [31], [32], [33]. Donor plasmid (“BE356”, ∼9 kB) was generated by modifying the 277.pCCLsin.cPPT.hPGK.eGFP.Wpre plasmid (a kind gift from L. Naldini, San Raffaele Telethon Institute for Gene Therapy, Milan, Italy) to contain a truncated eGFP that starts at nucleotide 37 of the coding sequence [25].

### mRNA synthesis

For mRNA synthesis *in vitro* transcription, capping and tailing were performed similar to Warren et al. [34]. First, GFP-ZFN2 and GFP-ZFN1, encoded on the SP202A and SP202B plasmids, were linearized with Xba I restriction enzyme; GFP-TALEN1 and GFP-TALEN2, encoded on M733L and M733R plasmids, were linearized with Afl II. T7 MEGAscript kit (Life Technologies, Grand Island, NY) was used with a reduced GTP concentration (1.5 mM) and 6 mM m^7^G(5')ppp(5')G RNA Cap Structure Analog (New England Biolabs, Ipswich, MA) added to synthesize RNA. Then the Poly(A) Tailing and MEGAClear kits (Life Technologies, Grand Island, NY) were used according to the manufacturer's directions.

### Transfection

The Neon transfection system (Life Technologies, Grand Island, NY) was used with 10 µl tips for all experiments unless indicated otherwise. Highly concentrated (> ∼0.8 µg/µl) transfection grade plasmids (e.g. Purelink HiPure) were used except as indicated in **Figure S1**. Plasmids in which eGFP is driven by a ubiquitous promoter were used in initial trials to identify a set of electroporation parameters suited for GS cells. Parameters that gave minimally ∼20% GFP+ following transfection of a ubiquitous GFP expression plasmid, with low to moderate toxicity based on qualitative assessments were pursued further and included 990/40/1, 1200/30/1 or 1400/20/1 (Voltage, Width, Pulse). A typical transfection included from 500 ng to 3 µg of DNA with from 0.5 to 3×10e5 cells plus 9 µl Buffer R/T (from Neon kit). For lipofection, Lipofectamine-2000 reagent was used to prepare DNA/lipid complexes according to the manufacturer's directions. DGC6 cells were trypsinized and plated in a well of MEFS just prior to applying DNA/lipid complexes at a ratio of 1 µg DNA or mRNA per 3×10e5 cells plated, the same ratio used in the Neon electroporations when lipofection and Neon were compared directly.

### Generation of gene-corrected cell lines

30 or 17 transfections were performed in serial, using MPG5 (passage 9) and F8R (passage 16) cells respectively, with each transfection containing 2×10e5 cells, 2.4 µg of donor plasmid (BE356) and 0.8 µg ZFN expression plasmid (M500). The Neon settings were 1400/20/1. Transfected cells were cultured and expanded for ∼4 weeks and subjected to flow sorting with Aria II (BD Biosciences, San Jose, CA) leading to the isolation of 110 GFP+ cells (MPG5 transfection) or 70 GFP+ cells (F8R transfection). In the case of MPG5-derived sorted cells the GFP+ gate was defined less stringently resulting in the presence of a contaminating GFP negative population; therefore a second sort was performed to further enrich for the GFP+ cells. The MPG5-derived sorted cells were named GT59 and were passaged altogether 21 times prior to transplantation. F8R-derived sorted cells were named GT65 and were passaged altogether 35 times, including modification with Histone-GFP lentivirus, prior to transplantation.

### Molecular analysis of gene-corrected cell lines

Genomic DNA was isolated from GT59 and GT65 cells using Dneasy Blood and Tissue kit (Qiagen, Valencia, CA). PCR analysis was performed using GoTaq polymerase (Promega, Fitchburg, WI) and the following primer pairs: DF5 (in *ROSA26* promoter) 5′-AAGTCGCTCTGAGTTGTTATCAGTA-3′ with DF2 (within mutational insert) 5′-GCTTCGGAGCCGCTTTAACCCA-3′ or with DF3b (flanking insert junction) 5′-CTTCACCTCGGCGCGGGTCT-3′; S11 (GFP, 5′ of insert) 5′-CTGACCCTGAAGTTCATCTGCACCAC-3′ with DF7 (GFP, 3′ of insert) 5′-GGTTCACCAGGGTGTCGCCCTCG-3′. Standard Sanger sequencing was used to analyze gel purified PCR products.

For quantitative RT-PCR analysis RNA extraction was performed using RNeasy Mini Kit with on column DNAse treatment (Qiagen, Valencia, CA) following the manufacturer's instructions. Reverse transcription was performed using qScript reverse transcriptase (Quanta Biosciences, Gaithersburg, MD). RNA samples were subjected to a negative control test in which reverse transcriptase was omitted. Quantitative PCR was performed using Perfecta SYBR Fast Mix Low Rox (Quanta Biosciences, Gaithersburg, MD) on a Stratagene MX3000P. Primer pairs used were as follows: ActbF 5′- GGCTGTATTCCCCTCCATC-3′, ActbR 5′- TGCCAGATCTTCTCCATGTC-3′, Pou5f1F 5′- CCTGCAGAAGGAGCTAGAACAGT-3′, Pou5f1R 5′-TGTTCTTAAGGCTGAGCTGCAA-3′, Gfra1F 5′- CACCCTGGATTTGCTGATGT-3′, Gfra1R 5′- AGTGTGCGGTACTTGGTGC-3′, Sall4F 5-CACGAAAGGCAACCTGAAG-3′, Sall4R 5′-ACGGAGATCTCGTTGGTCTT-3′, Sohlh1F 5′- CCCTGGATCCCTCACTCATG-3′, Sohlh1R 5′- GACCCACCAGGAACAATGTCA-3′. We verified that the amplified products were pure by evaluating a dissociation curve that was generated at the end of each PCR and by running the products on an agarose gel.

### Immunocytochemistry

GT59 or GT65 cells were cultured in a 96-well high-content imaging plate (BD Biosciences, San Jose, CA). Prior to ZBTB16 immunostaining cells were treated with 1 µM retinoic acid or vehicle control (0.02% ethanol) in SPGF medium for two (GT65) or three (GT59) days. Cells were washed twice with phosphate buffered saline (PBS), fixed in 4% paraformaldehyde for seven minutes (or in 10 minutes ice cold methanol for GFRA1), washed twice with PBS, permeabilized in PBT (PBS with 0.1% triton-x) for 15 minutes and blocked for one hour in 1X Blocking Reagent (Roche; Indianapolis, IN) diluted in PBS. For GFRA1 the permeabilization step was omitted and the blocking reagent was 10% donkey serum in PBS. Primary antibodies and dilutions used were: rabbit anti-DAZL (1∶1000, Ab34139), mouse anti-POU5F1 (1∶200, C-10; Santa Cruz Biotechnology, Santa Cruz, CA), mouse anti-ZBTB16 (1∶500, mAb 2A9; EMD Millipore, Billerica, MA), rabbit anti-SOHLH1 (1∶200, generous gift of A. Rajkovic) [35], goat anti-CDH1 (1∶500, AF748, R&D systems; Minneapolis, MN), goat anti-GFRA1 (1∶50, AF560; R&D systems, Minneapolis, MN), rabbit anti-ETV5 (1∶500, Ab102010; Abcam, Cambridge, MA) or rabbit anti-GFP (1∶1000, AB290, Abcam, Cambridge, MA). Primary antibodies were applied overnight at 4 degrees. Cells were washed with PBT except for GFRA1 immunostaining, where PBS was used instead. Secondary antibodies were Alexa-594- conjugated goat anti-rabbit or goat anti-mouse IgG (1∶500; Life Technologies, Grand Island, NY) or Cy3- conjugated donkey anti-goat (1∶500; Jackson Immuno Research, West Grove, PA). Secondary antibodies were diluted in PBT together with 1 µg/ml DAPI and were applied for two hours at room temperature or overnight at 4°C, followed by washing in PBT (or PBS for anti-GFRA1). All incubations and washes were carried out at room temperature unless specified otherwise. For a negative control the primary antibody was omitted. KIT immunostaining and analysis was performed as previously described [36].

### Transplantation

Transplantations into busulfan treated adult mice were carried out similarly to Ogawa et al. [37]. Eight week old athymic nude mice (CrTac:NCr-*Foxn1^nu^*; Taconic, Hudson, NY) were injected intraperitoneally with 35-38 mg/kg busulfan (Sigma, St. Louis, MO) and used for transplantation 7-17 weeks later. In certain experiments (where indicated) GT65 donor cells were treated with pLUG-H2B (Histone H2B-GFP) lentivirus and sorted for bright GFP+ cells prior to transplantation to enhance detection of the colonies [30]. We also performed transplantations into genetically sterile *Kit^W-v^*/*Kit^W^* pups [38]. 8 to 26 day old *Kit^W-v^*/*Kit^W^* recipients were obtained by breeding parental strains WB/ReJ *Kit^W^*/J and C57BL/6J-*Kit^W-v^*/J (The Jackson Laboratory, Bar Harbor, ME). To prepare the donor cells for transplantation, they were trypsinized together with MEFs, washed in media, filtered using a 40 µm cell strainer (BD Biosciences, San Jose, CA), counted with a hemocytometer to determine the germ cell concentration (MEFs and germ cells were distinguished based on size and morphology) and resuspended in media at 7–50×10^6^ germ cells/mL (see Table S1 for details). 7–10 µl were injected into adult testes or 2–3 µl into pup testes. Trypan blue (0.025%, Sigma, St. Louis MO)) was added just prior to loading the needle. Mice were given 1 mg/kg meloxicam (Butler Schein Animal Health, Melville, NY) analgesia sub-cutaneously, anesthetized by isoflurane inhalation or Nembutal injection (i.p. 80 mg/kg) and donor cells were injected into the rete testes through an efferent ductule. The efficiency of filling within the seminiferous tubules was visualized with trypan blue. Analyses were performed at least eight weeks post-transplantation. Testes with less than 50% fill (based on trypan blue visualization) or busulfan resistance (weighing more than 0.05 g) were not analyzed.

### Ethics Statement

This study was carried out in strict accordance with the recommendations in the Guide for the Care and Use of Laboratory Animals of the National Institutes of Health. The protocol was approved by the Bloomington Institutional Animal Care and Use Committee of Indiana University. Surgery was performed under Nembutal or isoflurane induced anesthesia and all efforts were made to minimize suffering. Cell lines and plasmids generated in this study will be made available upon request.

## Results

**Figure 1:**
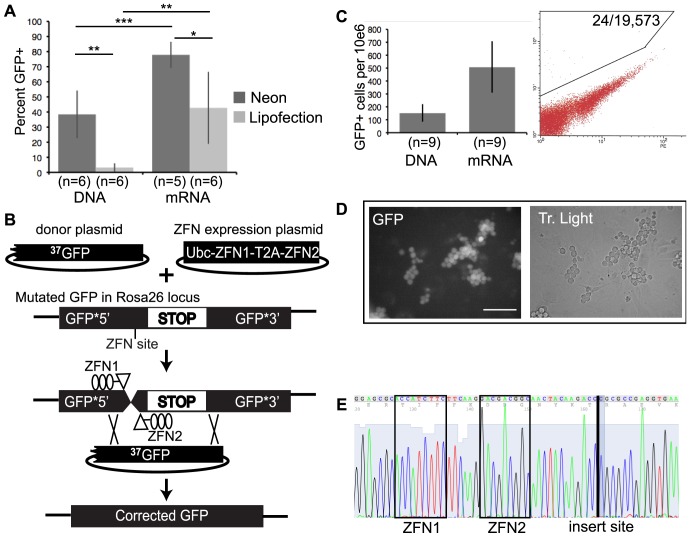
ZFN-mediated genome editing in GS cells. (A) Neon transfection (1200/30/1) was used to transfect 3×10e5 cells with 1.0 µg of em-GFP plasmid DNA (pCDNA6.2/emGFP) or 1.0 µg of capped and poly-adenylated mRNA coding for pmaxGFP and transfection efficiency was quantified by flow cytometry three days after transfection. Lipofectamine-2000 was used to transfect the same ratio of cells:DNA or cells:mRNA as in the Neon experiment. The mean and standard deviation of percentage of GFP+ cells from three experiments are shown. (*p<0.05, **p<0.01,***p<0.001, Student T test). (B) Schematic depicting the two plasmids used in genome editing experiments. The donor DNA (“^37^GFP”; plasmid BE356) contains a fragment of the GFP coding sequence lacking the first 37 nucleotides and serves as a donor template. “Ubc-ZFN1-T2A-ZFN2” (plasmid M500) contains a bicistronic expression cassette with a human Ubiquitin C promoter driving expression of two ZFNs directed to a recognition site in the GFP gene and separated by a T2A skip sequence. GS cell lines were derived from mice carrying a mutated GFP gene, with a 85 nucleotide stop codon and frame shift insertion (labeled “STOP”), introduced into the *ROSA26* locus by standard knockin technology [25]. (C) 0.8 µg of Ubc-ZFN1-T2A-ZFN2 (M500) plasmid or 0.8 µg each of synthesized mRNA ZFN1 and ZFN2, together with 2.4 µg donor plasmid (BE356), were transfected (1400/20/1) into MPG6 cells on day 1 and genome editing events were quantified on day 5 or 7 (data pooled). Histogram shows mean +/- standard error mean. The dot plot shows sample results of a single transfection of donor DNA and ZFN mRNAs. (D) GFP fluorescence (left) or corresponding transmitted light image in GT59 cells following two sorts to enrich for GFP+ cells. Bar represents 50 microns. (E) Chromatogram showing corrected GFP gene sequence of PCR amplified genomic DNA from GT59 cells. The ZFN recognition sites are indicated by boxes and the site in which the mutation was replaced by donor DNA is indicated by a line.

To develop genome editing technology in GS cells we sought an efficient mode of gene delivery because, similar to many primary cell types, GS cells are difficult to transfect [39]. As expected, lipofection of DNA was extremely inefficient. In contrast, relatively high transfection efficiency with good viability could be achieved using a Neon electroporator (**Fig. 1A** **and S1**). Optimization experiments, such as testing the effect of passage on transfection effeciency, were facilitated using the Neon system because it required fewer cells than other electroporators, a critical factor when working with cells that are challenging to isolate or culture (**Fig. S1**). Using the Neon system to introduce synthetic mRNA instead of plasmid DNA improved transfection efficiency even further, although lipofection of mRNA was also quite effective (**Fig. 1A** **and S1**).

### Genome editing in GS cells

We utilized a mouse model for a generic recessive disease in which a mutated GFP gene was integrated into the *Gt(ROSA)26Sor* (hereafter, *ROSA26*) locus [25]. We derived multiple GS cell lines from homozygous mutant GFP mice and used a set of previously described ZFNs and donor DNA, which serves as a template for repair of the DSB [25] (**Fig. 1B**). Expression of a pair of ZFNs from a single Ubiquitin C promoter was accomplished by separating the ZFN coding sequences by a T2A sequence, which causes “ribosomal skipping.” Flow cytometry was used to detect cells with the corrected GFP sequence based on fluorescence, providing a sensitive, quantitative readout for genome editing (**Fig. 1C**). We used the Neon to transfect GS cells with ZFN and donor DNA and in pilot experiments determined an ideal ratio of ZFN:donor DNA (data not shown) that could reproducibly lead to rare GFP+ cells; transfection of donor alone never yielded GFP+ cells likely because the regions of homology on the donor DNA were quite short (<1000 bp) compared to standard gene targeting vectors. Other attempts at optimization included testing the effect of modulating the cell cycle using dimethylenastron, an inhibitor of the mitotic kinesin Kif11 (Eg5); however, reproducible improvements were not observed (data not shown). In contrast, we did see modest improvement in genome editing when ZFNs were introduced in the form of synthetic mRNA (**Fig. 1C**). Increased editing was also observed when using TALENs instead of ZFNs to create a DSB (**Fig. S2**).

In order to establish a homogeneous population of gene-corrected cells we scaled up the transfection of ZFN and donor plasmid DNA, cultured the transfected cells for several weeks to allow the rare gene-corrected stem cells to expand in number and then isolated the gene-corrected cells by flow cytometry. This gene correction procedure was performed on two independently derived mutant GFP cell lines, ultimately leading to the generation of two GFP+ cell lines, GT59 and GT65 (**Fig. 1D** **and Fig. S2**).

### Molecular and phenotypic analysis of gene-corrected GS cells

The genomic DNA of GT59 and GT65 cells was PCR amplified using primers flanking the site of the mutation in GFP to analyze the molecular nature of the gene targeting event. A PCR product specific to the targeted cell lines was found to have the precise sequence expected for repair of the ZFN-induced DSB via homologous recombination with the donor DNA (**Fig. 1E** **and Fig. S2**). Despite the fact that over 90% of the GT59 and GT65 cells exhibited GFP fluorescence, the untargeted DNA could be PCR amplified along with the targeted DNA (**Fig. S2**). The results supported the idea that both cell lines had undergone monoallelic gene correction.

**Figure 2:**
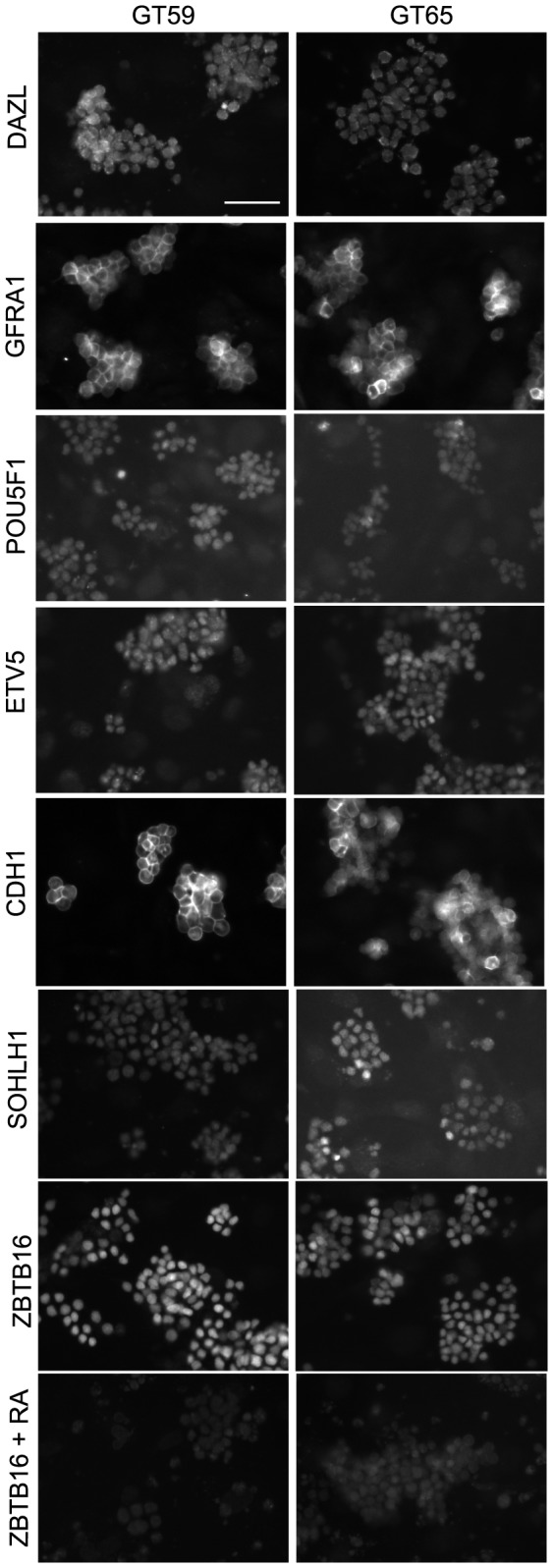
Retention of the spermatogonial phenotype following gene correction. Immunostaining was performed on gene-corrected GT59 (left) and GT65 cells (right): DAZL, a germ cell specific marker; GFRA1, POU5F1, ETV5, CDH1, and SOHLH1, markers of undifferentiated spermatogonia. Additionally, GT59 and GT65 cells were treated with the differentiation factor, retinoic acid (1 µM) or a vehicle control and then immunostained to examine levels of ZBTB16, a marker of undifferentiated spermatogonia. Bar represents 50 microns.

GS cells can be maintained in culture for multiple years while retaining functional stem cell activity [40]. Still, it was important to characterize the cellular phenotype of the gene-corrected cells because they had undergone multiple *in vitro* manipulations including transient exposure to ZFNs, which have the potential for off-target cutting. We performed immunocytochemistry on GT59 and GT65 cells and demonstrated expression of several spermatogonia-associated mRNAs and proteins including ZBTB16, POU5F1, GFRA1, ETV5, CDH1 and SOHLH1 (**Fig. 2** **and Fig. S3**). This expression profile is consistent with the known phenotype of typical GS cell cultures, which comprise SSCs and differentiating progenitor cells. It is also known that GS cells differentiate in response to retinoic acid, resulting in a reduction in ZBTB16+ cells (undifferentiated) and an increase in KIT+ cells (differentiated) [36]. We tested whether the gene-corrected GT59 and GT65 cells were responsive to retinoic acid. Indeed, GT59 and GT65 cells exhibited a reduction in ZBTB16 (Fig. 2) and an increase in KIT+ cells (Fig. S3 and data not shown) following retinoic acid treatment, suggesting that the cells could differentiate similar to unmodified cells (**Fig. 2**). Altogether the results showed that the gene-corrected cells shared many properties with unmodified cells: they expressed multiple markers of undifferentiated spermatogonia and were responsive to retinoic acid.

### Colonization by gene-corrected GS cells following transplantation

**Figure 3:**
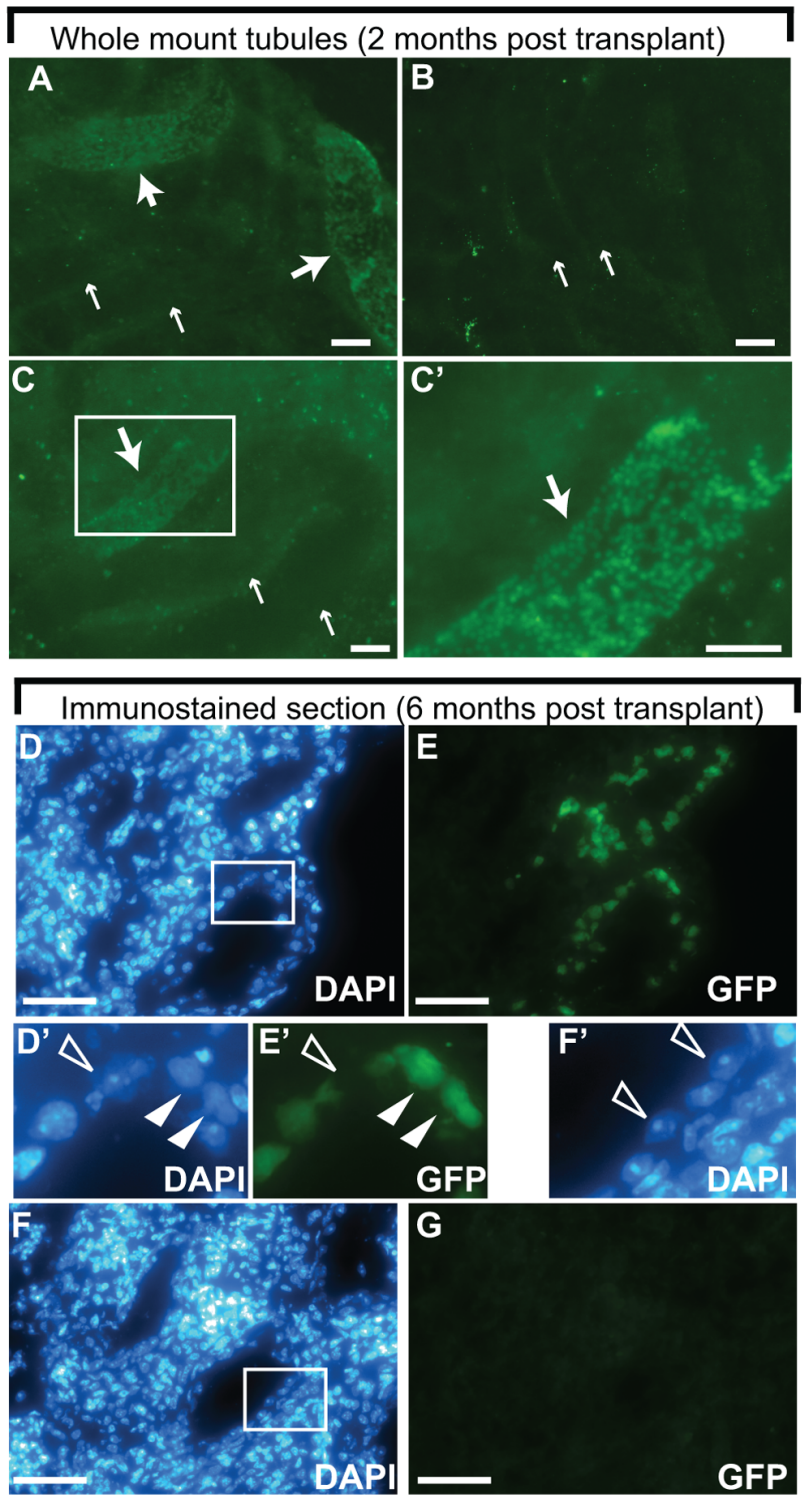
Colonization by gene-corrected GS cells following testicular transplantation. (A) Whole mounted squash preparation of seminiferous tubules depicting a seminiferous tubule (arrows) extensively colonized by gene-corrected GT59 SSCs two months following transplantation into a *Kit^W-v^*/*Kit^W^* sterile pup testis. (B) Non-transplanted control. (C) Whole mounted squash preparation of seminiferous tubules depicting a colony of GT65 SSCs two months following transplantation into a busulfan treated adult testis. Visualization of GT65 cells was facilitated by modification with Histone-GFP lentivirus prior to transplant. (A–C) Large arrowheads indicate GFP+ colonies and small arrows indicate autofluorescence in nearby tubules. Bar  = 100 microns. (C′) Higher magnification image of the boxed area in (C). Bar  = 50 microns. (D–G) Immunostaining with anti-GFP antibody (E, G, E′) or DAPI staining (D, F, D′, F′) of a cryosection of a GT59 colony 6 months following transplantation into *Kit^W-v^*/*Kit^W^* pup testis (D, E, D′, E′) or non-transplanted control testes (F, G, F′). Boxed area in D corresponds to the higher magnification view in D′ and E′. Triangles indicate donor-derived GFP+ cells. Boxed area in F corresponds to the higher magnification view in F′ and depicts the “Sertoli Cell Only” phenotype of non-transplanted *Kit^W-v^*/*Kit^W^* testes. The Sertoli cells are indicated by open triangles. Bar  = 25 microns.

Another characteristic of SSCs is their ability to home to a niche and colonize the seminiferous tubules following transplantation into testes of a pharmacologically or genetically sterilized mouse [4]. We used two well established transplantation models to test the colonization ability of the gene-corrected GS cells: busulfan treated immunocompromised adult mice and sterile *Kit^W-v^*/*Kit^W^* pups. Our goal was to perform a qualitative assessment of the developmental capacity of the gene-corrected cells following transplantation. Following transplantation of GT59 cells into busulfan treated adults we observed modest colonization (1–2 colonies per testes; Table S1) compared to published studies wherein typical colonization efficiency would be 30–50 colonies for the same number of cells injected [36], [41]; however, the results were confounded by the relatively weak GFP expression driven by the ROSA26 promoter in GT59 cells. In a second round of transplants into busulfan treated mice, this time using GT65 cells modified with a bright Histone-GFP reporter, modest colonization levels were also observed (**Fig. 3C** and Table S1). We also performed transplantations into sterile *Kit^W-v^*/*Kit^W^* pups because the microenvironment in pup testes can improve colonization efficiency compared to that of an adult [38]. The primary goal of the transplantations into sterile pups was to test whether the gene-corrected cells could restore fertility. Transplant recipients failed to sire progeny following several months of breeding tests (data not shown). Nonetheless, the testes of some *Kit^W-v^*/*Kit^W^* transplant recipients were evaluated for colonization by both whole mounting of seminiferious tubules and immunostaining of cross sections and colonization by donor cells was observed (**Table S1 and** **Fig. 3A–C**). In summary, GT59 and GT65 cells homed to the niche and formed colonies of proliferating spermatogonia, indicating that both cell lines retained some stem cell characteristics (**Fig. 3A–C** **and Table S1**) but cross sections of colonies did not contain spermatids (**Fig. 3D–G**). Whether the failure to produce sperm was a result of abnormalities in the transplanted cells or the recipient testes was unclear.

## Discussion

The main outcome of our study is demonstration of the use of ZFNs and TALENs to edit the genome in GS cells. Our results build on those of other studies in which gene correction rates were ∼0.2% in human ES cells or ∼1% in mouse ES cells [25], [42]. In SSCs previous studies of genome editing utilized conventional targeting methods without engineered nucleases [43], [44]. For instance, one study showed that following transfection of 2.4×10^8^ cells with a targeting vector containing large (>2000 bp) homology arms, antibiotic selection and PCR screening led to the isolation of two targeted cell clones. We obtained a ∼0.1% gene correction rate using engineered nucleases (Fig. 1C). Thus, the induction of a site specific double-strand break increased the targeting frequency by>100,000 fold. The low rate of gene correction in SSCs may reflect inherent mechanisms of genome protection unique to germ cells; intrinsic differences in efficiency between cell types are not unexpected [25]. Fortunately, one could invoke a strategy to enrich for corrected cells using multiple published methods. Enrichment strategies include FACS purification of transfected cells [45], the purification of cells that have undergone correction based on the modification of a surrogate reporter which dramatically enriches for modified cells [46], and the use of donor constructs and designs containing selectable markers that allow one to select for modified cells using the selectable marker and then subsequent “scarless” elimination of the selectable marker after identification [47]. Thus, the low frequency of gene correction in SSCs does not preclude genome editing from being accomplished in this important stem cell type.

The implications of our study are multi-faceted with applications in research and potentially therapeutics. Much progress remains to be made in understanding mechanisms controlling SSC fate, particularly in humans. The ability to make precise modifications to the genome could facilitate analysis of gene function, thereby advancing our understanding of SSCs and spermatogenesis. For instance, a point mutation identified in a genome-wide association study to be potentially associated with spermatogenic failure could be directly tested for functional importance using the technology demonstrated here. The ability to generate fluorescent reporters of gene expression by targeted addition is another potential research application. The relevance of these applications extends even beyond the study of SSCs, given that upon testicular transplantation of genetically engineered GS cells new transgenic mouse models can be generated [39].

The implications of our study for medicine are two-fold. First, we addressed a pervasive challenge in gene therapy, namely gene delivery in a “hard to transfect” primary-like stem cell. Gene delivery is a particularly significant issue for nuclease-mediated gene correction because it is necessary to introduce three components into cells (e.g. two ZFNS + donor). In this study we demonstrated a gene delivery approach that may be widely applicable to other stem cells. In our preliminary experiments adeno-associated virus (AAV6), and integration deficient lentivirus both were inadequate for accomplishing genome editing. Identification of a virus with the appropriate tropism for a cell type of interest and production of sufficient titers of infectious virus are among the complications of viral delivery. In contrast, following brief optimization experiments, we found the Neon electroporator could impart unprecedented high transfection rates with GS cells. Further, the approach can be applied on both a small and large scale, allowing for cell-type specific optimization experiments. Finally, by transfecting synthetic mRNA instead of plasmid DNA further improvements in gene delivery were attained. Delivering nucleases in the form of mRNA is particularly attractive because it ensures that the potentially toxic nucleases are present only transiently.

Moving beyond the issue of gene delivery, our study demonstrated proof of concept for the process of *ex vivo* gene therapy, wherein a patient's cells are isolated, genetically modified *in vitro* and then reintroduced into the patient. Admittedly, challenges must be overcome before translation to the clinic. A feature critical to the success of *ex vivo* gene therapy is availability of a culturing method, something that had been established for rodent SSCs, but that is an area of fervent yet controversial investigation for human SSCs [8], [9], [48], [49], [50]. Regarding correction of genes other than GFP, other examples of genome engineering at a model target locus have generally proven to be highly relevant in predicting applicability to new loci. In the case of genetic defects causing spermatogenic failure, corrected SSCs would have a selective advantage and may not need to be enriched before transplantation as only corrected SSCs would produce sperm. For diseases in which corrected cells do not have a selective advantage, strategies described above could facilitate enrichment of rare cells with the corrected loci.

Many genetic diseases affect cells or tissues for which a cognate stem cell type is unknown and or impossible to culture. Many diseases also are systemic in nature, affecting numerous cell types that cannot be treated with a single cell type. Furthermore, even for certain diseases of the blood that have been successfully treated by genetic modification and transplantation of autologous hematopoietic stem cells, patients then live with the burden of potentially passing the heritable trait on to their children. Ultimately the most permanent and all-encompassing cure of a genetic disease would be to genetically modify the germ cells.

The use of SSCs in our study is especially unique because unlike all other adult stem cell types, SSCs are capable of generating sperm, which carry genetic information to the next generation. SSCs are also unique in their developmental plasticity, capable of transdifferentiation to multiple lineages or even to pluripotency without the use of exogenous genetic factors {Kanatsu-Shinohara, 2004 #28;Simon, 2009 #15;Zhang, #43}. An obvious application one could imagine is treatment of infertility caused by mutations affecting germ cell development. However, several substantial issues would need to be addressed before applying germline gene therapy in humans. First, our transplantation study revealed that the gene-corrected cells appeared to exhibit attenuated differentiation capability. The cause of this shortcoming is unclear. Previous studies showed that GS cells can be transfected and passaged extensively *in vitro* while still retaining the ability to produce spermatozoa following transplantation [39], [40]. Our data did not distinguish among potential effects of technical issues with the transplant recipients, genetic or epigenetic effects on the cells unrelated to genome editing, or unintended genomic changes related to off-target cutting by the ZFNs. In other experiments we have found that delivering ZFNs (both these and others) via DNA expression vectors causes gross chromosomal rearrangements (MP, unpublished data). Additionally, when the nucleases are delivered as mRNA or when TALENs or CRISPR/Cas9 nucleases are used instead of ZFNs genomic toxicity is reduced. Thus, in the future targeted modification of SSCs should probably be performed using mRNA delivery of TALENs or CRISPR/Cas9. Finally, deep consideration of the ethical consequences would be warranted before modifying the genome of human germ cells for what would certainly be controversial purposes of transgenerational gene therapy.

## Acknowledgments

Many thanks to C. Heim, Y. Zheng and J. Balke for technical assistance, B. Ellis for the donor plasmid, J. Powers and the Light Microscopy Imaging Center and C. Hassel and the Flow Cytometry Core Facility for instrumentation.

## Supporting Information

Figure S1 **Optimization of gene delivery in GS cells.** (**A**) Neon transfection (1400/20/1) was used to transfect 3×10e5 cells with 0.8 µg of em-GFP plasmid DNA (pCDNA6.2/emGFP) or 0.8 µg of capped and poly-adenylated mRNA coding for pmaxGFP and transfection efficiency was quantified by flow cytometry 4 hours, 24 hours or 72 hours after transfection. The mean and standard deviation of percentage of GFP+ cells for duplicate transfections in a representative experiment are shown. (**B and C**) Neon transfection (1200/30/1) was used to transfect 3×10e5 cells with 1.0 µg of em-GFP plasmid DNA (pCDNA6.2/emGFP) and transfection efficiency was quantified by flow cytometry one, three or seven days after transfection. The mean and standard deviation of percentage of GFP+ cells for duplicates in two experiments are shown in (**B**). The mean and standard deviation of Y-mean (GFP) signal intensity are shown from one representative experiment in (**C**). (**D**) Neon transfection (1200/40/1) was used to transfect 5×10e4 wildtype GS cells (DGC1 cell line derived from DBA/2 mice (Dann et al., 2008)) with 145 ng GFP expression plasmids (prepared by Qiagen Spin Miniprep) on day 1 and flow cytometry was used to quantify transfection efficiency on day 4. In each plasmid GFP was driven by a different promoter: CMV (cytomegalovirus enhancer/promoter; plasmid M171), CMV-CBA (cytomegalovirus enhancer, chicken b-actin promoter; plasmid A633), EF1a (elongation factor 1 a promoter; plasmid A491)and Ubc (Ubiquitin C promoter; plasmid M279). The reduced transfection efficiency in (**D**) compared to other figures is likely caused by the lower quality of miniprep DNA and lower quantity of cells and DNA used in this experiment. (**E**) 1.0 µg of HiPure em-GFP plasmid DNA (pCDNA6.2/emGFP) was transfected (1200/30/1) into 3×10e5 low passage (P4 and P7) or high passage (P29 and P32) DGC6 wildtype cells on day 1 and GFP was quantified with a FACSCalibur on day 4 (n = 4 each, 2 experiments combined). (*p<0.05, Student T test).
(EPS)

Figure S2 **Optimization and molecular analysis of genome editing in GS cells.** (**A**) 0.8 µg each of synthesized mRNA coding for ZFN1 and ZFN2, or TALEN1 or TALEN2, together with 2.0 µg donor plasmid (BE356), were transfected (990/40/1) on day 1 and genome editing was quantified on day 4 (n = 4 each, 2 experiments combined). Both histograms display the mean and standard error mean. (**B**) Flow cytometry analysis of GT59 cells following sorting and *in vitro* expansion of gene-corrected cells. Dot plots show GFP on the y-axis and orange autofluorescence on the x-axis. (**C**) Schematic depicting the primers used for amplification of genomic DNA from gene-corrected cells. Primer 1 is in the *ROSA26* promoter region, primer 4 is in the 5′ region of GFP, primer 2 is in the mutational insert within the GFP coding sequence, primer 3 spans the junction of the mutational insert and GFP coding sequence, and primer 5 is in the 3′ portion of GFP. (**D**) PCR products with various primer combinations using genomic DNA isolated from cells before targeting (“pre”; MPG4 cell line) or GT59 cells after the first sort (“post1”) or GT59 cells after the second sort (“post2”). The doublet of PCR products amplified with primers 4 and 5, corresponding to the mutated and gene-corrected alleles, are indicated by a box. The products of this PCR reaction were separated by gel electrophoresis, cut out and purified to obtain two distinct products for sequencing. The sequence of the bottom (gene-corrected) band is shown in Figure 1. Identical results were obtained with PCR analysis of genomic DNA from GT65 cells.
(EPS)

Figure S3 **Phenotypic characterization of gene corrected cells.** (A) Gel analysis of quantitative RT-PCR products following 40 cycles of amplification of the indicated mRNAs from GT59 and GT65 cells. Lanes showing products of reactions without reverse transcriptase are indicated by “RT-.” (B) Average cycle threshold (“Ct”) values (n = 2 technical duplicates) from the indicated qRT-PCR reactions. (C) Left: Forward/side scatter dot plot of GT59 cells showing the R1 gate used for analysis. Right: Histogram depicting PE fluorescence (isotype control or KIT expression) in GT59 cells immunostained with PE conjugated KIT antibody or isotype control. The plot overlays the data from cells treated with retinoic acid or vehicle control for two days. (D) Histogram depicting the mean and standard deviation of percentage KIT+ staining in GT59 cells treated with retinoic acid or vehicle control for two days (n = 2 for each treatment).
(EPS)

Table S1 **Colonization analysis of whole tubules from transplanted testes.**
(DOC)
