# Supplementary material for: Genome Editing in Mouse Spermatogonial Stem/Progenitor Cells Using Engineered Nucleases
Source: PLoS One. 2014 Nov 19;9(11):e112652. doi: 10.1371/journal.pone.0112652 (PMC4237364; doi:10.1371/journal.pone.0112652)
Supplement: Table S1 — Colonization analysis of whole tubules from transplanted testes. (DOC) [file pone.0112652.s004.doc]

Supplementary Table S1

Colonization analysis of whole tubules from transplanted testes

| Donor cells | Recipient type | Time post transplant (months) | Cell concentration  (10e6 cells/mL) | Total testes analyzed | Testes with colonies | Total colonies |
| --- | --- | --- | --- | --- | --- | --- |
| GT59 | Busulfan | 2 | Low (5-8) | 2 | 2 | 3 |
| GT65 | Busulfan | 2 | Low (5-8) | 5 | 1 | 2 |
| GT59 | Busulfan | >4 | Low (5-8) | 4 | 0 | 0 |
| GT65+H2B-GFP | Busulfan | 2 | Low (5-8) | 5 | 5 | 8 |
|  |  |  |  |  |  |  |
| GT59 | *Kit* | 2-3 | Low (5-8) | 2 | 2 | 7 |
| GT59 | *Kit* | >4 | Low (5-8) | 3 | 0 | 0 |
| GT59 | *Kit* | >4 | High (>23) | 3 | 2 | 9 |
| GT65 | *Kit* | >4 | High (>23) | 2 | 1 | 1 |
